# A conserved CENP-E region mediates BubR1-independent recruitment to the outer corona at mitotic onset

**DOI:** 10.1016/j.cub.2024.01.042

**Published:** 2024-02-13

**Authors:** Jeraldine Weber, Thibault Legal, Alicia Perez Lezcano, Agata Gluszek, Calum Paterson, Susana Eibes, Marin Barisic, Owen R. Davies, Julie P.I. Welburn

**Affiliations:** 1 Wellcome Trust Centre for Cell Biology, School of Biological Sciences, University of Edinburgh, Edinburgh EH9 3BF, Scotland, UK; 3 Now in Department of Anatomy and Cell Biology, Faculty of Medicine and Health Sciences, 3640 University street, McGill University, H3A0C7, Montreal, Quebec, Canada; 4 Cell Division and Cytoskeleton, Danish Cancer Institute, Strandboulevarden 49, 2100, Copenhagen, Denmark; 5 Department of Cellular and Molecular Medicine, Faculty of Health and Medical Sciences, University of Copenhagen, 3C Blegdamsvej, 2200 Copenhagen N, Denmark

**Keywords:** motor, mitosis, microtubule

## Abstract

The outer corona plays an essential role at the onset of mitosis by expanding to maximize microtubule attachment to kinetochores^1, 2^. The low-density structure of the corona forms through the expansion of unattached kinetochores. It comprises the RZZ complex, the Dynein adaptor Spindly, the plus-end directed microtubule motor CENP-E, and the Mad1/Mad2 spindle assembly checkpoint proteins^3–10^. CENP-E specifically associates with unattached kinetochores to facilitate chromosome congression^11–16^, interacting with BubR1 at the kinetochore through its C-terminal region (2091-2358)^17–21^. We recently showed that CENP-E recruitment to BubR1 at kinetochores is both rapid and essential for correct chromosome alignment. However, CENP-E is also recruited to the outer corona by a second slower pathway that is currently undefined^19^. Here, we show that BubR1-independent localization of CENP-E is mediated by a conserved loop that is essential for outer corona targeting. We provide a structural model of the entire CENP-E kinetochore targeting domain combining X-ray crystallography and Alphafold2. We reveal that maximal recruitment of CENP-E to unattached kinetochores critically depends on BubR1 and the outer corona, including Dynein. Ectopic expression of the CENP-E C-terminal domain recruits the RZZ complex, Mad1, and Spindly, and prevents kinetochore biorientation in cells. We propose that BubR1-recruited CENP-E, in addition to its essential role in chromosome alignment to the metaphase plate, contributes to the recruitment of outer corona proteins through interactions with the CENP-E corona-targeting domain to facilitate the rapid capture of microtubules for efficient chromosome alignment and mitotic progression.

## Results and discussion

CENP-E is essential for the alignment of chromosomes in metaphase. However, the mechanisms underlying CENP-E recruitment to unattached kinetochores remain unclear. Here, we define the two major molecular pathways for CENP-E targeting to kinetochores and provide mechanistic insights into how CENP-E connects to its kinetochore cargo.

### Dynein mediates CENP-E recruitment to and removal from kinetochores

**Figure 1:**
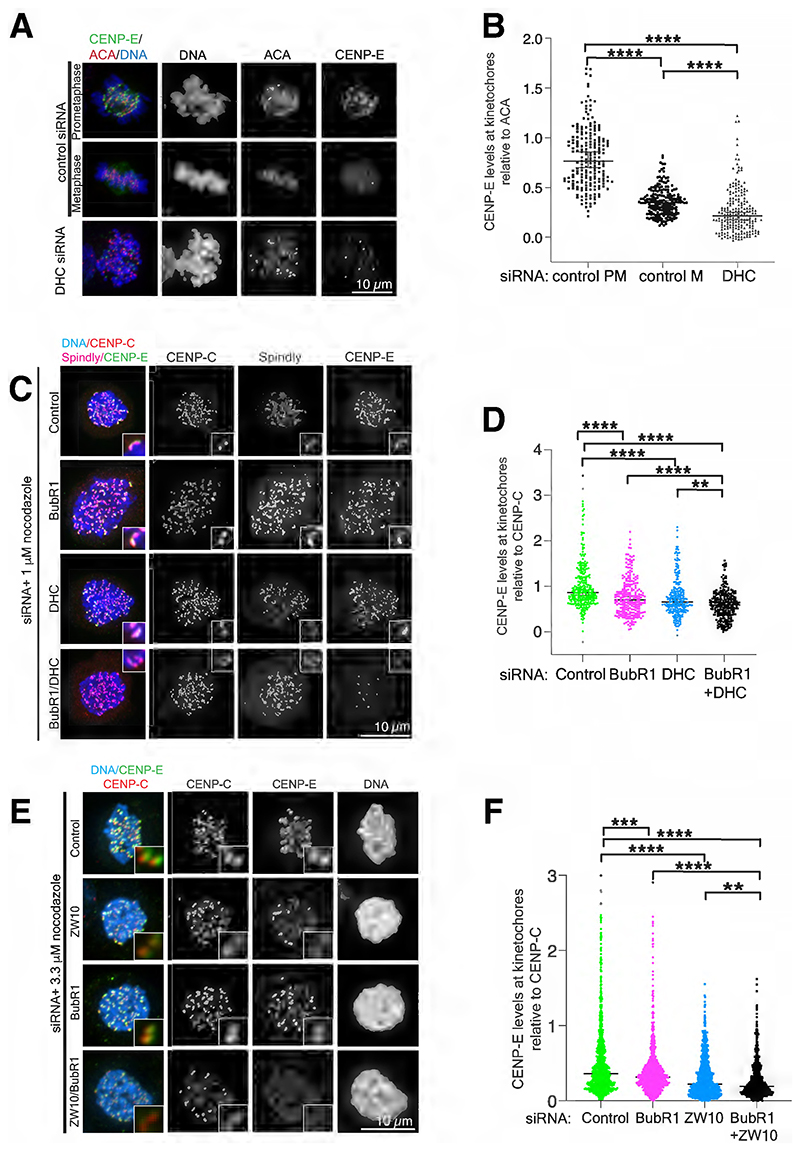
CENP-E recruitment to unattached kinetochores depends on two distinct pathways involving BubR1 and Dynein. (A) Representative immunofluorescence images of HeLa cells in prometaphase and metaphase after depletion with control siRNA, or after depletion with Dynein Heavy chain (DHC) siRNA. The cells were stained for endogenous CENP-E, ACA and DNA. (B) Scatter dot plot showing quantification of CENP-E intensity relative to ACA for cells in prometaphase (PM) and metaphase (M) after treatment with control or DHC siRNA for 48 hours. n=201, 202 and 200 kinetochores respectively. Median and 95% confidence interval are presented. Asterisks indicate ordinary Kruskal-Wallis test significance value. ****P<0.0001. (C) Representative immunofluorescence images of HeLa cells after siRNA depletion of BubR1, Dynein Heavy Chain (DHC) or BubR1/DHC, or control siRNA and treated with 1 μM nocodazole for 2 hours. The cells were stained for endogenous CENP-E, Spindly, CENP-C and DNA. (D) Scatter dot plot showing quantification of normalized CENP-E intensity relative to CENP-C after control, BuBR1, DHC and BubR1/DHC siRNA depletion. Median and 95% confidence interval are presented. Each point represents the intensity of CENP-E over CENP-C at one kinetochore. Asterisks indicate Kruskal-Wallis test significance value. ****P<0.0001, **P=0.01. n=310, 250, 270 and 270 kinetochores for control, BubR1, DHC and BubR1/DHC siRNA depletion respectively. Experiments were repeated three times. Also see Figures S1C-E. (E) Representative immunofluorescence images of HeLa cells after siRNA depletion of BubR1, ZW10 or BubR1/ZW10, or control siRNAs and treated with 3.3 μM nocodazole for 15 hours. The cells were stained for endogenous CENP-E, CENP-C and DNA. (F) Scatter dot plot showing quantification of normalized CENP-E intensity relative to CENP-C after control, BubR1, ZW10 and BubR1/ZW10 siRNA depletion. Median and 95% confidence interval are presented. Each point represents the intensity of CENP-E over CENP-C at one kinetochore. Asterisks indicate Kruskal-Wallis test significance value. ****P<0.0001, **P=0.001, **P=0.04. n=1188, 914, 1025 and 820 kinetochores for control, BubR1, ZW10 and BubR1/ZW10 siRNA depletion respectively. The experiment was repeated three times. Scalebars: 10 μm. Also, see Figures S1F-G.

Since our previous data indicated that CENP-E targeted to kinetochores independently of BubR1^19^ and CENP-E is present at the outer corona^6,8^, we examined which outer corona components are linked to CENP-E dynamics at the kinetochore. While the RZZ complex (composed of Rod-ZW10-Zwilch) and Spindly are essential for the self-assembly of the outer corona, Dynein strips the outer corona, including CENP-E, once kinetochore-microtubule attachments are stabilized^22–24^. Thus we hypothesized that CENP-E would be enriched at kinetochores upon Dynein depletion. To test this, we depleted the Dynein Heavy Chain (DHC) and examined CENP-E levels at kinetochores. Unexpectedly, upon DHC depletion, CENP-E levels at kinetochores were strongly reduced compared to CENP-E levels at attached kinetochores in wild-type prometaphase and metaphase cells, indicating that Dynein plays a role in CENP-E recruitment to kinetochores (Figures 1A, B).

To further dissect the relationship between Dynein and CENP-E, we targeted endogenous CENP-E by treating cells with the allosteric inhibitor GSK923295. This treatment led to loss of CENP-E at kinetochores and co-localization with centrin, indicating that the protein had moved to spindle poles (Figures S1A, B). However, DHC depletion led to loss of CENP-E accumulation at mitotic spindle poles. Some residual CENP-E was observed close to spindle poles, possibly due to inhibited CENP-E being moved by the poleward flux of microtubules, or incomplete depletion of DHC^25^ (Figures S1A, B). A parallel independent study reported the CENP-E pole accumulation was Dynein- and flux-dependent^26^. Overall, these observations confirm that Dynein is important for kinetochore localization of CENP-E and CENP-E kinetochore stripping to the poles.

Dynein is also present at unattached kinetochores, but is not required for expansion of the outer corona^8,27^. To further characterize the role of Dynein in CENP-E kinetochore recruitment prior to microtubule attachment, we depleted either BubR1, to disrupt CENP-E recruitment to the outer kinetochore^19^, or Dynein, or both in cells treated with nocodazole to depolymerize microtubules. Depletion of BubR1 alone decreased CENP-E levels at unattached kinetochores, in agreement with previous work (Figures 1C, D)^18,19,28^. Depletion of DHC also reduced CENP-E levels at kinetochores (Figures 1C, D). Depletion of both led to a strong reduction in CENP-E at unattached kinetochores (Figures 1C, D), with residual CENP-E possibly due to incomplete depletion of BubR1 and DHC (Figures S1C-E).

We next tested how disrupting outer corona assembly by disrupting the RZZ complex assembly impacts CENP-E kinetochore targeting. Despite increasing the nocodazole concentration to 3.3 μM, we could not reproducibly observe crescent-shaped structures, possibly due to our cell line. Depletion of ZW10, a core component of the RZZ complex essential for outer corona assembly, compromised CENP-E recruitment to unattached kinetochores, while co-depletion of both BubR1 and ZW10 further reduced CENP-E targeting (Figures 1E, F). Some residual CENP-E remained at kinetochores, perhaps due to residual BubR1 and ZW10 protein (Figuress S1F, G). Overall, our data indicate that both Dynein and the RZZ complex are essential for targeting CENP-E to unattached kinetochores during prometaphase.

### Distinct CENP-E domains mediate outer corona targeting and BubR1-binding

**Figure 2:**
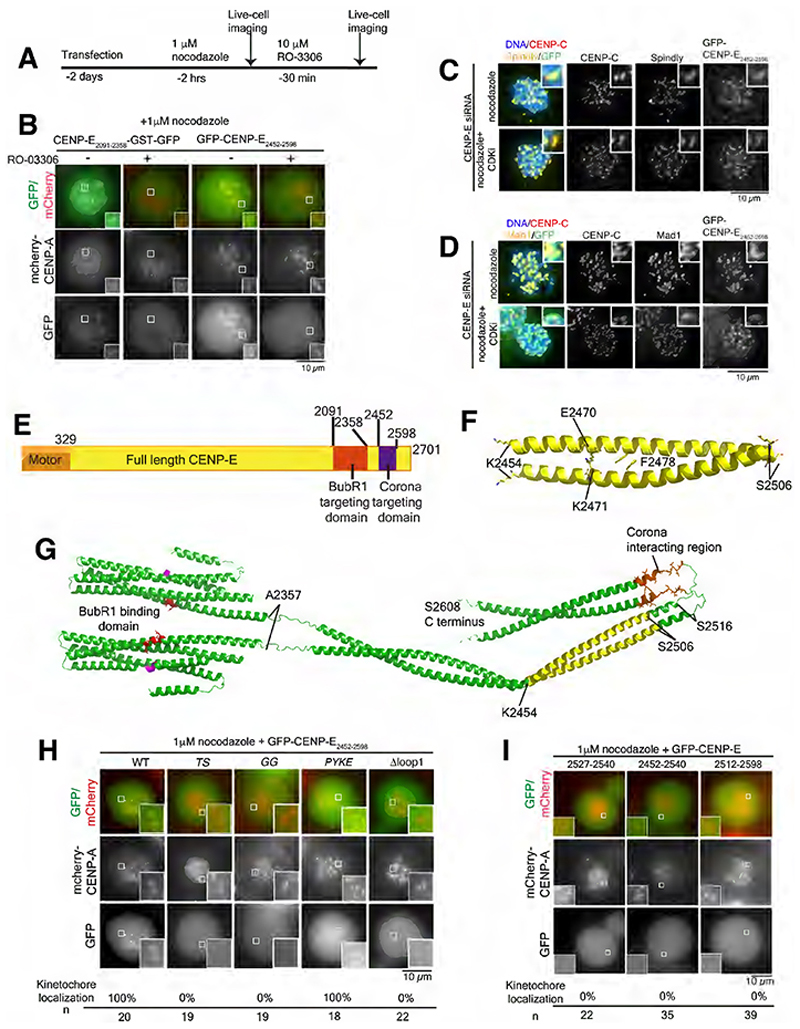
A conserved loop in CENP-E, distinct from the BubR1-binding domain, is essential for targeting to the outer corona. (A) Schematic timeline of the experiment represented in B. (B) Representative images of live HeLa cells stably expressing mCherry-CENP-A transfected with CENP-E_2091-2358_-GST-GFP and GFP-CENP-E_2452-2598_, treated with 1 μM nocodazole for 2 hours and imaged. The cells were then incubated with 10 μM CDK1 inhibitor RO-3306 for 30 minutes before imaging again. Experiments were repeated twice. Also see Figure S2A. (C, D) Representative immunofluorescence images of HeLa cells transfected with GFP-CENP-E_2452-2598_ after CENP-E depletion by siRNA and treated with 3.3 μM nocodazole and CDK1 inhibitor RO-3306, stained for CENP-C and either Spindly (C) or Mad1 (D). Also see Figure S2B, C. (E) Schematic diagram of CENP-E, highlighting the motor (pale orange) and kinetochore- and corona-targeting domains (bright orange and purple respectively). (F) X-ray crystallography-derived structure of CENP-E_2454-2506_ in cartoon representation. The conserved amino acids making the coiled-coil interface are represented in ball and stick mode (PDB:8OWI). Also see Table S1. (G) Model of the CENP-E_2055-2608_ generated by combining Alphafold2 models of its monomeric kinetochore targeting domain and dimeric outer corona targeting domain. The conserved residues 2527-2538 essential for corona-targeting are in stick and ball orange. The domain crystallized (F) is painted yellow. The residues essential for interaction with BubR1 are in red and the conserved DFSE motif is painted magenta. See Figures S2F and S3. (H, I) Top, representative live-cell images of HeLa cells expressing mCherry-CENP-A and transfected with GFP-CENP-E point (H) or domain (I) mutants and treated with 1 μM nocodazole for 2 hours. Bottom, quantification of the kinetochore localization of GFP-CENP-E domain mutants related to images above. Experiments were repeated twice or three times. Scalebars: 10 μm.

Previous work identified CENP-E_2055-2608_ as the kinetochore-targeting domain of CENP-E, which is dominant negative^17,19^. Our data indicate that CENP-E recruitment to kinetochores is dependent on BubR1 through the minimal BubR1-interacting domain (CENP-E residues 2091-2358)^19^ and on the corona-localized Dynein and the RZZ complex (Figure 1). We thus hypothesized that CENP-E_2055-2608_ contains an outer corona-targeting domain that is separate from the minimal BubR1-interacting domain. Briefly, CDK1 inhibition causes disassembly of the outer kinetochore, including the KMN network which recruits BubR1, but leaves the outer corona of kinetochores intact^8,9^. We predicted that CENP-E_2091-2358_ would associate with unattached kinetochores but its kinetochore localization would disappear upon CDK1 inhibition-induced outer kinetochore-disassembly. An outer corona-targeting domain of CENP-E would, in contrast, remain associated with unattached kinetochores after CDK1 inhibition. To test this, we expressed different CENP-E domains in a HeLa cell line expressing mCherry-CENP-A to mark kinetochores, and treated the cells with nocodazole to induce unattached kinetochores. We imaged the transfected cells before and after incubation with the CDK1 inhibitor RO-3306 using live-cell imaging (Figure 2A). The minimal BubR1-interacting CENP-E domain, expressed as a dimer, CENP-E_2091-2358_-GST-GFP, localized to nocodazole-treated unattached kinetochores but disappeared after treatment with the CDK1 inhibitor, indicating the ability of CENP-E_2091-2358_ to localize to kinetochores is dependent on the outer kinetochore integrity as we predicted (Figures 2B, S2A)^19^. GFP-CENP-E_2452-2598_ localized to the outer kinetochore both in the presence and absence of the CDK1 inhibitor, although its levels were decreased by 2-fold in part due to photobleaching during the experiment (Figures 2B, S2A). These results indicate GFP-CENP-E_2452-2598_ associates with the outer corona. We then imaged GFP-CENP-E_2452-2598_ after depletion of endogenous CENP-E. GFP-CENP-E_2452-2598_ associated with corona crescents, co-localizing with Spindly and Mad1 (Figures 2C, D). We could occasionally observe the outer corona (marked by Spindly), detaching from kinetochores marked by CENP-C, with GFP-CENP-E_2452-2598_ co-localizing with Spindly (Figures S2C, S2D). Additionally BubR1 depletion did not reduce CENP-E_2452-2598_ levels at kinetochores (Figures S2E, F). Overall, these results reveal that CENP-E_2452-2598_ associates with the outer corona.

### A flexible loop linker is essential for CENP-E outer corona targeting

Next, we sought to determine the structure of this minimal corona-targeting domain (Figure 2E). This domain contains a highly conserved region from Xenopus to humans that is predicted unstructured and flanked by two predicted α-helices (Figure S2G). We therefore recombinantly expressed and purified CENP-E_2452-2598_ (Figure S2H).

To define whether CENP-E_2452-2598_ is dimeric, we used crosslinking with the Bis(sulpho-succinimidyl)suberate (BS3) crosslinker, which reacts with primary amines at the N termini of proteins and with lysine side chains within a 11.4 Å range. Using this approach, we found that CENP-E_2452-2598_ forms a dimer (Figure S2H). We obtained protein crystals that diffracted anisotropically with resolution limits between 2.14 Å and 2.71 Å (Table S1). Structural determination revealed a parallel dimeric coiled-coil in which only the first helical region of the construct was observed in electron density (Figure 2F), likely because the construct underwent C-terminal degradation during crystallization. The resultant structural model corresponds to amino acids K2454 to S2506 of CENP-E. The structure reveals a tight left-handed coiled-coil in which the hydrophobic heptad interface involves aromatic stacking of F2478 residues. Surrounding interactions that stabilize the coiled-coil include salt bridges between the E2470 and K2471 side chains (Figure 2F). The dimeric structure is consistent with the behaviour of CENP-E_2452-2598_ in solution (Figure S2H), and is supported by previous data indicating this region mediates dimerization of the kinetochore-targeting domain of CENP-E_2055-2608_^19^.

To gain insights into the broader architecture of the corona and kinetochore-targeting domains, we built a molecular model of the CENP-E_2055-2608_ dimer (Figure 2G). Alphafold2 predicted two distinct domains, in keeping with our previous findings^19^. As the BubR1-targeting domain and corona-targeting domain are monomeric and dimeric, respectively^19^, we used Alphafold2 to model these regions separately in their respective oligomeric states. We then combined these models by connecting two predicted BubR1-targeting domain monomer structures to models of the corona-targeting domain dimer and the full C-terminal region of CENP-E. We first used Alphafold2 to model a region encompassing the BubR1-targeting domain (amino-acids 2055-2377) as a monomer. This model revealed a four-helical bundle structure with high confidence and the BubR1 binding region is solvent-exposed (Figures 2G, S3A-C). The highly conserved sequence “DFSE” may also contribute to BubR1 binding (Figure 2G, magenta). We then used Alphafold2 to model the subsequent region encompassing the corona-targeting domain (amino-acids 2356-2608) as a dimer, using our newly solved crystal structure as a template. All models predicted two consecutive parallel homodimeric coiled-coils (residues 2370-2515 and 2537-2598) that flank the central unstructured region (2516-2532) (Figure 2G). Alphafold2 modeled residues 2531-GGGSGIV-2537 as helical or unstructured (orange, Figures 2G, S3A-C) with low confidence. Residues 2527-2538 form a highly conserved patch and contribute to the loop and beginning of the helix (Figure S2G). The structure we determined is part of the coiled-coil on the N-terminal side of the loop and the conserved patch (highlighted in yellow, Figures 2F, G). We combined these structures to present a model of the complete CENP-E_2055-2608_ dimer. This model fits with the rotary shadowing of CENP-E_2055-2608_, which revealed the presence of a globular domain adjacent to an extended domain, with total lengths of 36 nm and 40 nm, respectively (Figure 2G)^19^.

Since residues 2527-2538 are highly conserved across multiple species (Figures 2G, S2F), we hypothesized that this region could be important for the recruitment of CENP-E to the outer corona. Mutating T2529 and S2534 to AA (*TS*), or G2532 and G2533 to NE (*GG*) in CENP-E_2452-2598_ abolished their recruitment to the corona (Figure 2H). However, mutating highly conserved residues 2452-2455 (PYKE) (Figure S2G), did not disrupt kinetochore targeting (Figures 2H, S2D). We generated a mutant lacking the conserved residues 2527-2538, in the loop and the start of the consecutive helix (GFP-CENP-E Δloop1). GFP-CENP-E Δloop1 showed no localization to the outer corona, indicating this conserved part of the loop is critical for recruitment during mitosis (Figures 2H, S2D). We next tested if the conserved loop sequence was sufficient for outer corona targeting by transfecting GFP-CENP-E_2527-2540_ into HeLa cells. The sequences _2527_PLTCGGGSGIVQNT_2540_, the _2452_helix-loop_2540_ and _2512_loop-helix_2598_ were not sufficient for targeting to the outer kinetochore (Figure 2I). We also made a mutant predicted to lack the loop whilst retaining the coiled-coil (Figure S3D). GFP-CENP-E Δloop2 (2513-2537 deleted) also failed to localize to kinetochores (Figure S3E). Overall, the CENP-E conserved patch and flanking helical coiled-coils are essential for outer corona targeting.

### BubR1 and the corona-associated proteins target CENP-E to kinetochores

**Figure 3:**
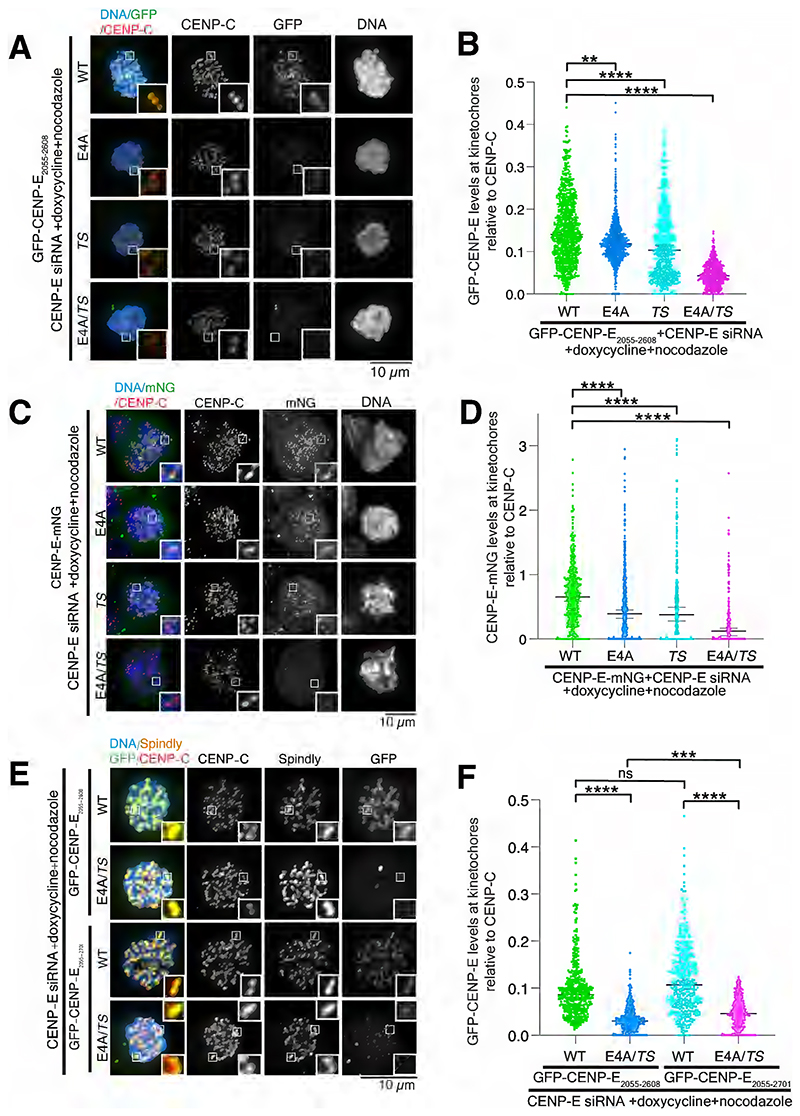
Disruption of the BubR1-binding region and corona-targeting loop prevents CENP-E localization to kinetochores. (A) Representative immunofluorescence images of stable Flp-in HeLa cells expressing GFP-CENP-E_2055-2608_ wild type and mutants, treated with 3.3 μM nocodazole after CENP-E siRNA-depletion, stained for CENP-C and DNA. Protein expression was induced by adding 1 μg/ml doxycycline for 48 hours. Related to Figure S4A. (B) Scatter plot showing the ratio of fluorescence intensity of GFP-CENP-E_2055-2608_ intensity relative to CENP-C for CENP-E wild type and mutants at kinetochores (kinetochore numbers for WT n=864, E4A n=673, *TS* n=667 and E4A *TS* n=704). The experiment was repeated 3 times. Bars represent median and 95% confidence interval. **** P<0.0001, ** P<0.01. Asterisks indicate ordinary Kruskal-Wallis test significance value. (C) Representative immunofluorescence images of U2OS cells expressing full-length CENP-E-mNG wild type and mutants after CENP-E siRNA-depletion treated with 3.3 μM nocodazole, stained for CENP-C and DNA. Protein expression was induced by adding 1 μg/ml doxycycline for 24 hours. Related to Figure S4B. (D) Scatter plot showing the ratio of fluorescence intensity of mNG relative to CENP-C for CENP-E wild type and mutants at kinetochores (kinetochore numbers for WT n=400, E4A n=487, *TS* n=270 and E4A *TS* n=184). Bars represent the median and 95% confidence interval. Asterisks indicate ordinary Kruskal-Wallis test significance value, with **** P<0.0001. (E) Representative immunofluorescence images showing the localization of stably-expressed GFP-CENP-E_2055-2608_ and GFP-CENP-E_2055-2701_ in HeLa Flp-in cells after CENP-E siRNA-depletion, and treatment with 3.3 μM nocodazole. Protein expression was induced by adding 1 μg/ml doxycycline for 48 hours. Related to Figure S4A. (F) Scatter plot showing quantification of GFP-CENP-E_2055-2608_ and GFP-CENP-E_2055-2701_ intensity relative to CENP-C at kinetochores in HeLa Flp-in cells related to panel (E). For cells expressing GFP-CENP- E_2055-2608_ WT and E4A *TS*, n=1373 and n=1336 kinetochores. For cells expressing GFP-CENP-E_2055-2701_ WT and E4A *TS*, n=1299 and n=1178 kinetochores. Bars represent median and 95% confidence interval. **** P<0.0001. Asterisks indicate ordinary Kruskal-Wallis test significance value. The experiment was repeated three times. Scalebars: 10 μm.

Next we hypothesized that disrupting both BubR1 and corona-binding in CENP-E should completely disrupt recruitment to unattached kinetochores. We disrupted the acidic patch using the previously published GFP-CENP-E_2055-2608_ E4A mutant^19^ and the corona-targeting loop by mutating T2529 and S2534 to AAs (*TS*). As expected, GFP-CENP-E_2055-2608_ localized to unattached kinetochores whilst the GFP-CENP-E_2055-2608_ E4A and GFP-CENP-E_2055-2608_ *TS* mutants targeted there to lesser levels (Figures 3A, B). Kinetochore localization of the double mutant GFP-CENP-E_2055-2608_ E4A *TS* was almost completely abolished (Figures 3A, B).

Next, we tested the recruitment of CENP-E to unattached kinetochores, in the context of the full-length protein. Endogenous CENP-E was depleted in stable U2OS cell lines inducibly expressing full-length CENP-E-mNG WT, E4A, *TS* or E4A *TS* (Figure S4B). CENP-E-mNG E4A and CENP-E-mNG *TS* showed a marked reduction in kinetochore localization, while CENP-E-GFP E4A *TS* was not present at unattached kinetochores (Figures 3C, D).

Finally, farnesylation of the C terminus of CENP-E has been proposed to be essential for formation of the outer corona^29^. Our previous work has implicated the last 100 amino acids of CENP-E binds to PRC1-crosslinked microtubules rather than kinetochores^30^. Thus we examined the contribution of the CENP-E C terminus to CENP-E binding to kinetochores and the outer corona assembly in stable Flp-in HeLa cell lines inducibly expressing GFP-CENP-E_2055-2608_ and GFP-CENP-E_2055-2701_ (Figure S4A). After depletion of endogenous CENP-E, GFP-CENP-E_2055-2608_ and GFP-CENP-E_2055-2701_ were both equally recruited to unattached outer kinetochores, with the GFP signal co-localizing with Spindly at the outer corona (Figures 3E, F). There was a significant decrease in levels of the double mutants compared to the levels of respective GFP-CENP-E WT at kinetochores (Figures 3E, F). There was also more residual GFP-CENP-E_2055-2701_ E4A *TS* at kinetochores compared to GFP-CENP-E_2055-2608_ E4A *TS*. The last 100 amino acids may therefore modestly contribute to outer corona binding. Overall, our data identify two major parallel pathways that recruit CENP-E to unattached kinetochores.

### CENP-E_2055-2608_ relocalizes corona components and inhibits kinetochore biorientation

**Figure 4:**
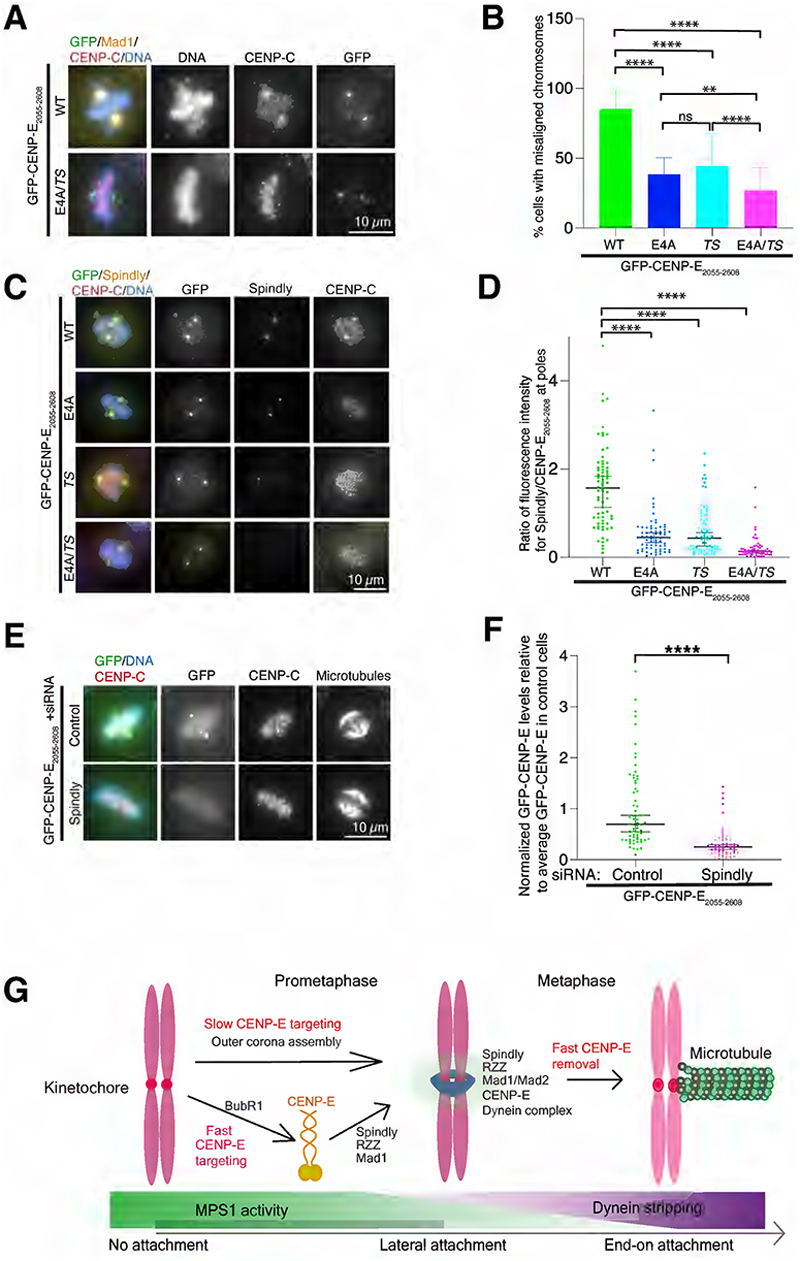
CENP-E_2055-2608_ associates with Spindly, Mad1, and the RZZ complex through the corona-binding motif. (A) Representative immunofluorescence images of HeLa cells transfected with GFP-CENP-E_2055-2608_ wild type and mutants, stained for Mad1, CENP-C and DNA. (B) Bar graph showing percentage of misaligned chromosomes in the presence of GFP-CENP-E_2055-2608_ (n=61), GFP-CENP-E_2055-2608_ E4A (n=52), GFP-CENP-E_2055-2608_ *TS* (n=63) and GFP-CENP-E_2055-2608_ E4A *TS* (n=56), data from 5 experiments. Mean and standard deviation are presented. Asterisks indicate ordinary one-way ANOVA test significance value. ****P<0.0001. (C) Representative immunofluorescence images of HeLa cells transfected with GFP-CENP-E_2055-2608_ wild type and mutants, stained for Spindly, CENP-C and DNA. (D) Scatter plot showing the ratio of fluorescence intensity of Spindly to GFP-CENP-E_2055-2608_ wild type or mutants at spindle poles (WT n=70, E4A n=70, *TS* n=74 and E4A *TS* n=64). Bars represent median and 95% confidence interval. Kruskal-Wallis test with P-value ***<0.0001 (P=0.0340). The experiment was repeated 3 times. Related to Figure S4C-G. (E) Representative immunofluorescence images showing the localization of stably-expressed GFP-CENP-E_2055-2608_ in HeLa Flp-in cells after control or Spindly siRNA-depletion. Protein expression was induced by adding 1 μg/ml doxycycline for 48 hours. (F) Scatter dot plot showing quantification of normalized GFP-CENP-E_2055-2608_ intensity around spindle poles relative to average GFP-CENP-E_2055-2608_ fluorescence intensity in control-treated cells (n=66) and in cells treated with siRNA-depleted Spindly (n=56) in HeLa Flp-in cells related to panel (E). Unpaired T-test with P ***<0.0001. Data from 2 independent experiments. Related to Figure S4H. (G) Schematic proposed model explaining the two modes of recruitment of CENP-E to kinetochores in early mitosis and CENP-E removal in metaphase. Scalebars: 10 μm.

GFP-CENP-E_2055-2608_ accumulates at spindle poles, due to the absence of microtubule plus-end activity^6,17,19^. This causes chromosome misalignment of a fraction of chromosomes, with 85.3% of cells expressing the domain displaying misaligned chromosomes (Figures 4A, B) ^17,19^. The majority of chromosomes would align and biorient, marked by the reduction of the spindle checkpoint protein Mad1 (Figures 4A, S4C). 38.5 and 44.4% of cells expressing either GFP-CENP-E_2055-2608_ E4A or GFP-CENP-E_2055-2608_*TS* displayed misaligned chromosomes, with chromosomes accumulating proximal to the poles (Figure 4B). In the presence of GFP-CENP-E_2055-2608_ E4A *TS*, 26.8% chromosomes remained aligned (Figures 4A, B). We did not arrest the cells in metaphase, thus our quantification underestimates the fraction of cells that aligned their chromosomes successfully in contrast to Cmentowski et al^31^.

We hypothesized that GFP-CENP-E_2055-2608_ binds other components of the detachable corona, that also co-accumulate at spindle poles and prevent chromosome congression. Indeed, in the presence of transiently-expressed GFP-CENP-E_2055-2608,_ Spindly, ZW10 and Mad1 were enriched at the spindle poles (Figures 4C, D, S4C-F). However, in the presence of GFP-CENP-E_2055-2608_ *TS*, there was a reduction in the fluorescence intensity of Mad1, Spindly and ZW10 at the poles (Figures 4C, D, S4C-F). There was also a reduction of corona proteins in the presence of GFP-CENP-E_2055-2608_ E4A, suggesting this mutant may have a long-range effect on the recruitment of corona proteins, possibly through BubR1’s ability to interact with other kinetochore proteins^32^. Finally Mad1, ZW10 and Spindly largely did not co-localize with GFP-CENP-E_2055-2608_ E4A *TS* (Figures 4C, D, S4C-F). In these experiments, we imaged GFP-CENP-E_2055-2608_ WT and mutants with similar levels (Figure S4G). Residual outer corona proteins were occasionally observed at kinetochores, reflecting some kinetochores may still be laterally attached. Antibody sensitivity varied between Spindly, Mad1 and ZW10. In total, these data indicate that GFP-CENP-E_2055-2608_ recruits components of the outer corona, including Mad1, Spindly and the RZZ complex. GFP-CENP-E_2055-2608_ mutants still accumulated at the poles (Figure 4) possibly through centrosome binding^19^, or heterodimerization with the endogenous CENP-E. Finally, Spindly siRNA-depletion dramatically reduced GFP-CENP-E_2055-2608_ at the poles in GFP-CENP-E_2055-2608_-expressing Flp-in cells (Figures 4E, F, S4H), indicating Spindly is important for CENP-E_2055-2608_ accumulation to the poles likely through dynein (Figures S1A, B).

Overall, our findings suggest the recruitment of corona-localized proteins to CENP-E_2055-2608_ at pole-proximal kinetochores, prevents the alignment of these chromosomes. These kinetochores may remain at the poles because CENP-E_2055-2608_ outcompetes full-length CENP-E. Yet these kinetochores may still bind Dynein through Spindly-associated CENP-E_2055-2608_ (Figures 4E, F), which would favor their minus-end accumulation^22,23,26^. It is also possible that CENP-E_2055-2608_ titrates the corona-localizing proteins away from kinetochores, which could prevent correct outer corona assembly at kinetochores and prevent their alignment.

Our work reveals that CENP-E is recruited to unattached kinetochores through at least 2 pathways: a BubR1-dependent pathway involving the domain 2055-2358 and a pathway dependent on the outer corona and Dynein involving domain 2452-2598 (Figure 4G). This was also reported in an independent study^31^. The BubR1 recruitment pathway is kinetically fast, acting within minutes and is essential for chromosome alignment, while the second pathway is slower^19^. The expansion of the outer corona, facilitated by Mps1 phosphorylation, needs to take place before CENP-E binds. CENP-E relocalizes the components of the outer corona Spindly, Mad1 and the RZZ complex, suggesting they interact directly or indirectly. We reveal that Dynein also unexpectedly plays a role in stabilizing CENP-E at the outer corona. We show here that adjacent, but structurally distinct, domains in CENP-E facilitate protein interactions for each pathway. However the two domains, through their interaction partners, may cause some crosstalk to facilitate CENP-E recruitment to kinetochores. BubR1-localized CENP-E could recruit and stabilize the outer corona proteins, further promoting CENP-E accumulation to the outer corona (Figure 4G). Future studies are required to identify the molecular basis for CENP-E recruitment to the outer corona and how the force produced by the motor activity of CENP-E allows chromosome alignment.

## STAR Methods

### Contact for Reagent and Resources

#### Lead contact

Further information and requests for resources and reagents should be directed to and will be fulfilled by the Lead Contact, Julie Welburn (Julie.welburn@ed.ac.uk).

#### Material availability

Plasmids and cell lines generated in this study can be obtained through the lead contact. The plasmids are listed in Table S2.

### Experimental Model and Subject Details

HeLa cells (93021013, Sigma Aldrich, RRID: CVCL_0030) were used and maintained in DMEM (Gibco) supplemented with 10% Tet-free FBS (A4736401, ThermoFisher), 5% Penicillin/Streptomycin (Gibco) and 2.5 mM L-Glutamine at 37°C in a humidified atmosphere with 5% CO_2_. Cells were checked monthly for mycoplasma contamination (MycoAlert detection kit, Lonza). HeLa LacZeo/TO Flp-In cells (kind gift from Stephen Taylor, University of Manchester, Manchester, UK) were used according to the Flp-In system protocol (Thermo Fisher). Cells were maintained in 8 μg/ml blasticidin and 4 μg/ml Zeocin.

*E. coli* BL21-CodonPlus (DE3)-RIL was used for recombinant protein expression.

### Method details

#### Cloning

To assay the localisation of CENP-E subdomains in cultured cells, constructs were generated from CENP-E transcript variant 1 (NM_001813.2) and cloned into pBABE-puro containing an N-terminal GFP tag or pcDNA5 FRT/TO after fusion with GFP, using restriction enzymes ^33^. Mutagenesis was performed using the Quickchange site-directed mutagenesis kit (200523, Agilent) according to the manufacturer’s guidance. Full-length CENP-E-mNeonGreen constructs in pcDNA5 FRT/TO were assembled with the assistance of the Edinburgh Genome Foundry, a synthetic biology research facility specialising in the assembly of large DNA fragments at the University of Edinburgh. DNA fragments were synthesized by Geneart. The full-length CENP-E constructs were then cloned into pENTR-GFP (IPC0793-Ian Chambers) and then subcloned into pLenti CMV/TO-DEST (gift from E. Campeau, Addgene plasmid #17291) ^34^ by LR recombination (Invitrogen) according to manufacturer’s instructions. Plasmids generated and used in this study are summarized in Table S2.

#### Cell culture and experiments

Stable clonal HeLa cells lines expressing mCherry-CENP-A were generated using a retroviral system as described previously ^33^. HeLa cells expressing mCherry-CENP-A were grown in DMEM (Life Technologies) supplemented with 10% FBS and Penicillin-Streptomycin (Gibco) at 37°C and 5% CO_2_. Cells were checked monthly for mycoplasma contamination (MycoAlert detection kit; Lonza). For live imaging, cells were plated on 35-mm glass-bottom microwell dishes (Ibdi). Transient transfections were conducted using Effectene reagent (QIAGEN) according to the manufacturer’s guidelines. Cells were imaged/fixed 24 to 48 hours later.

Gene knockdown was achieved by transfecting 60 pmol siRNA (Dharmacon, Thermo Fisher Scientific) using Lipofectamine RNAiMax (Thermo fisher) according to the manufacturer’s instructions. Cells were fixed 72 hours later. The siRNA sequences used were: 5’-AAGGAUCAAACAUGACGGAAUUU-3’ for DHC and 5′-GCAATCAAGTCTCACAGAT-3’ for BubR1 ^35^. For CENP-E and ZW10, sequences were 5’-AAGGUACAAUGGUACUAUAUUU-3’ and 5’-UGAUCAAUGUGCUGUUCAAUU-3’ respectively^8,18^. For Spindly, 5’-GAAAGGGUCUCAAACUGAAUU-3’^9^. The non-targeting control siRNA was 5’-UGGUUUACAUGUCGACUAA-3’. Treatment with GSK923295 was performed for 30 minutes at a concentration of 200 nM (Selleckchem). To depolymerize microtubules and create unattached kinetochores, cells were treated with 1 μM nocodazole. To generate detachable kinetochore crescents, cells were treated with 3.3 μM nocodazole (Calbiochem) overnight followed by addition of 10 μM RO-3306 (Bio-Techne Ltd) for 30 minutes before fixing (Pereira et al, 2018; Sacristan et al, 2018).

HeLa Flp-In cell lines expressing GFP-CENP-E_2055-2608_ and GFP-CENP-E_2055-2701_ mutants were generated using HeLa LacZeo/TO cells according to the Flp-In system protocol (Thermo Fisher). GFP-CENP-E_2055-2608_ was cloned into pcDNA5 FRT/TO vector. The plasmids were transfected and integrated using a Flp recombinase-mediated integration. During and after selection, the cell lines were maintained in DMEM supplemented with tetracycline-free FBS (Gibco), with 8 μg/ml blasticidin and 200 μg/ml hygromycin B. Expression of GFP-CENP-E constructs were induced with 1 µg/ml doxycycline for 48 hours (Sigma Aldrich).

To generate cells with inducible expression of full-length CENP-E variants, we performed lentiviral transduction in U2OS cell line with integrated Tet repressor (TetR) ^26^. Following infection, cells were selected using puromycin, and serial dilutions were performed for clonal selection.

#### Immunofluorescence, imaging and quantification

Cells were fixed with 3.8% formaldehyde in PHEM buffer (60 mM Pipes, 25 mM HEPES, 10 mM EGTA, 2 mM MgSO4, pH 7.0) or ice-cold methanol for 20 minutes and stained using rabbit anti-Spindly (1:200, Bethyl lab A301-354A), anti-Mad1 clone BB3-8 (1:1000, Merck), anti-ZW10 (1:100, Rabbit, Abcam ab21582), guinea pig anti-CENP-C (1:1000, MBL PD030) and mouse CENP-E (1:200, Abcam ab5093) antibodies. Hoechst 33342 (ThermoFisher Scientific; H3570) was used to stain DNA.

Images were recorded using a Deltavision core microscope (Applied Precision) equipped with a CoolSnap HQ2 CCD camera or widefield Eclipse Ti2 (Nikon) microscope equipped with a Prime 95B Scientific CMOS camera (Photometrics), using a 100x objective (CFI Plan Apochromat Lambda, 1.49 N.A). For fixed samples, Z-sections were acquired at 0.2-µm step intervals using the 100X objective lens. For live-cell imaging, 10 Z-sections were acquired at 0.5-µm step size using 100X objective lens. Cells were imaged in Leibowitz L15 medium (ThermoFisher Scientific). Images were stored and visualized using an OMERO.insight client (OME) ^36^. For quantification of protein levels, all images of each immunostaining experiment were acquired with identical illumination settings. Mean kinetochore fluorescence intensity within a circular ROI with an 8-pixel diameter was measured for CENP-C and the background intensity in an adjacent cytoplasmic area was also recorded. The ROIs were then used to record the fluorescence intensity of other proteins at kinetochores. Relative values for each kinetochore were calculated by subtracting the background values. They were then divided by the background corrected CENP-C signal for that kinetochore. For quantification of pole accumulation of CENP-E, mean pole fluorescence intensity, marked by the bipolar CENP-E_2055-2608_ localization within a circular ROI was measured and the background intensity in an adjacent cytoplasmic area was recorded. Average intensity was calculated by subtracting the background values. For ZW10, Mad1 and Spindly intensity, the ROIs defined in the CENP-E-stained channel were used, and average intensity was calculated as above. In the case of DHC depletion, if the poles were split, the two brightest poles were quantified using an ROI around centrin and transposing the ROI to the channel imaging CENP-E. For the quantification of chromosome alignment, chromosomes that were away from the metaphase plate, or within 2 μm from the CENP-E_2055-2608_ marked poles were classified as misaligned. Data were analysed using OME^36^ or ImageJ^37^. Images for immunofluorescence were deconvolved using softWoRx (Applied Precision) or AutoQuant (Media Cybernetics).

#### Protein expression and crystallization

His_6_-CENP-E_2452-2598_ was cloned into pET3aTr. For expression, *E. coli* BL21-CodonPlus (DE3)-RIL were transformed with the plasmid. Cultures were induced with 0.5 mM IPTG at OD_600_=0.6, overnight at 18°C. Cells were re-suspended in lysis buffer (50 mM HEPES pH 7.5, 500 mM NaCl, 40 mM imidazole, 1 mM EDTA, 5 mM β-Mercaptoethanol) supplemented with 1 mM PMSF and cOmplete EDTA-free protease inhibitor cocktail (Roche) and lysed by sonication. The lysate was cleared by centrifugation (50 minutes, 22,000 RPM) in a JA 25.50 rotor (Beckman Coulter), filtered and loaded onto a HisTrap HP column (GE Healthcare). His_6_-CENP-E_2452-2598_ was eluted in elution buffer (lysis buffer with 500 mM imidazole). The his-tag was cleaved with a 3C protease overnight. The sample was then applied to a HisTrap HP column after dialysis. CENP-E_2452-2598_ was collected in the flow-through and then concentrated and loaded on a Superdex 200 Increase 10/300 GL (GE Healthcare) pre-equilibrated in size-exclusion chromatography buffer (20 mM HEPES pH 7.5, 300 mM NaCl, 1 mM EDTA, 1mM DTT).

His_6_-CENP-E_2452-2598_ was concentrated to 11 mg/ml and crystallized in 0.2M Potassium citrate tribasic monohydrate, 20% w/v polyethylene glycol 3350 pH8.3 within a few days at room temperature. The crystal was protected in 30% glycerol mixed with mother liquor and frozen in liquid nitrogen.

#### Data collection and structure determination

X-ray diffraction data were collected at 0.9795 Å, 100 K, as 3600 consecutive 0.10° frames of 0.050 s exposure on a Dectris Eiger2 XE 16M detector at beamline I04 of the Diamond Light Source synchrotron facility (Oxfordshire, UK). Data were indexed, integrated in XDS^38^, scaled in Aimless^39^, and merged with anisotropic correction and ellipsoidal truncation by STARANISO (Tickle, 2018), using AutoPROC^40^. Crystals belong to tetragonal spacegroup P4_2_2_1_2 (cell dimensions a = 84.02 Å, b = 84.02 Å, c = 65.02 Å, α = 90°, β = 90°, γ = 90°), with a CENP-E dimer in the asymmetric unit. Structure solution was achieved through fragment-based molecular replacement using ARCIMBOLDO_LITE^41^ in which six helices of 14 amino-acids were placed by PHASER^42^ and extended by tracing in SHELXE utilizing its coiled-coil mode^43^. A correct solution was identified with a SHELXE correlation coefficient of 56.4%. Model building was performed through iterative re-building by PHENIX Autobuild^44^ and manual building in Coot^45^. The structure was refined using PHENIX refine^44^, using isotropic atomic displacement parameters with one TLS group per chain. The structure was refined against data to anisotropy-corrected data with resolution limits between 2.14 Å and 2.71 Å, to R and R_free_ values of 0.2161 and 0.2455 respectively, with 100% of residues within the favored regions of the Ramachandran plot (0 outliers), clashscore of 1.05 and overall MolProbity score of 0.81 ^46^. PDB entry is: 8OWI.

#### Structural modelling

Models were generated using a local installation of *Alphafold2*^47, 48^. The BubR1-targeting domain (amino-acids 2055-2377) was modelled as a monomer using the monomer pipeline. The corona-targeting domain (amino-acids 2356-2607) was modelled as a dimer using the multimer pipeline, with the newly solved crystal structure (PDB accession 8OWI) specified as a template. The resultant structures were aligned based on their overlapping sequences, and were joined to form two full chains, and their intervening linkers were fitted using geometry and Ramachandran restraints. *Alphafold2* multimer modelling data were analysed using modules from the ColabFold notebook^49^. Models were edited, combined and flexible linkers were remodelled using the *PyMOL* Molecular Graphics System, Version 2.0.4 Schrödinger, LLC, and *Coot*^45^.

#### Protein crosslinking

Recombinant his_6_-CENP-E_2452-2598_ and BS3 (Thermo Fisher Scientific) were mixed in a 1:1 ratio (w/w) for 30 minutes at room temperature. His_6_-CENP-E_2452-2598_ was used at 1mg/ml. The reaction was quenched with 200mM ammonium bicarbonate. The reaction was resolved by SDS–PAGE (4–12% Bis-Tris NuPAGE, Invitrogen) gel separation and stained using Instant Blue (Expedeon).

#### Quantification and statistical analysis

Statistical analyses were performed using Prism 9 (GraphPad Software). For the calculation of the error on the median, we report the upper 95% confidence interval. No statistical method was used to predetermine sample size. No samples were excluded from the analyses. The investigators were not blinded to allocation during experiments and outcome assessment. All experiments were performed and quantified from 2-4 independent experiments and representative data are shown. Sample sizes, statistical tests and p values are indicated in the figures and figure legends where relevant. We used p < 0.05 as the threshold for statistical significance and indicated in the figure and figure legend if the p-value was lower than 0.05, 0.01, 0.001 and 0.0001 using asterisks (^∗^or ^∗∗^or ^∗∗∗^, respectively).

#### Western blots

Western blots were performed using the antibodies listed in the table below.

## Supplementary Material

Supplementary Material

## Data Availability

Coordinates for the model of the X-ray structure of CENP-E_2454-2506_ and electron density maps have been deposited in the Protein Data Bank (PDB) under accession number 8OWI. They are publicly available as of the date of publication. Accession numbers are also listed in the key resource table. The paper does not report any original code. Any additional information required to reanalyze the data reported in this paper is available from the lead contact upon request.
