# Supplementary material for: A conserved CENP-E region mediates BubR1-independent recruitment to the outer corona at mitotic onset
Source: Curr Biol. Author manuscript; Available in PMC 2025 Jul 29. (PMC7617963; doi:10.1016/j.cub.2024.01.042)
Supplement: Supplementary Material [file EMS206577-supplement-Supplementary_Material.pdf]

Current Biology, Volume *34*

Supplemental Information

A conserved CENP-E region mediates BubR1-independent recruitment to the outer corona at mitotic onset

Jeraldine Weber, Thibault Legal, Alicia Perez Lezcano, Agata Gluszek, Calum Paterson, Susana Eibes, Marin Barisic, Owen R. Davies, and Julie P.I. Welburn

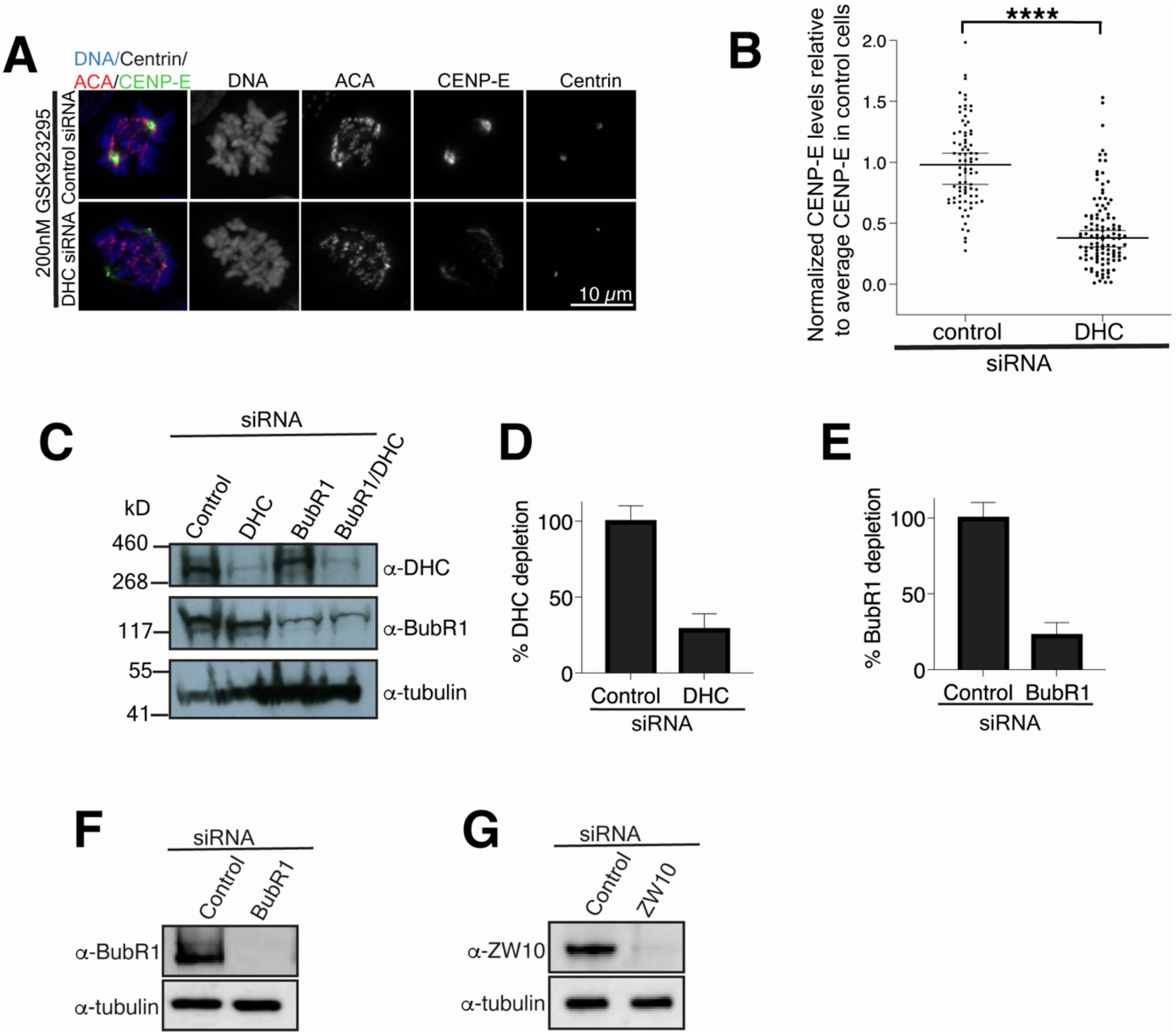

Figure S1. Dynein is required for spindle pole accumulation of CENP-E. Related to Figure 1. (A) Representative immunofluorescence images of HeLa cells after depletion with control or Dynein Heavy Chain siRNA. Cells were treated with 200 nM GSK923295 for 30 minutes, then stained for ACA, CENP-E and DNA. Scalebar: 10 μm. (B) Scatter dot plot showing quantification of normalized CENP-E intensity around spindle poles relative to average CENP-E levels for cells treated with 200 nM GSK923295 for 30 minutes. Median and 95% confidence interval are shown, n=83 and 113 cells measured respectively. For each cell, the intensity around both spindle poles marked by centrin was measured and averaged. Asterisks indicate Mann-Whitney test significance value. ****P<0.0001. (C) Western blot for cells in Figure 1C after siRNA treatment probed for BubR1/DHC and Tubulin as a loading control. (D, E) Quantification of the levels of depletion of DHC and BubR1 for blot in S1C. Error bars represent the mean and standard deviation. (F) Western blot for cells after control and BubR1 siRNA treatment probed for BubR1. (G) Western blot for cells in Figure 1E after control and ZW10 siRNA treatment probed for ZW10. Tubulin is presented as a loading control.

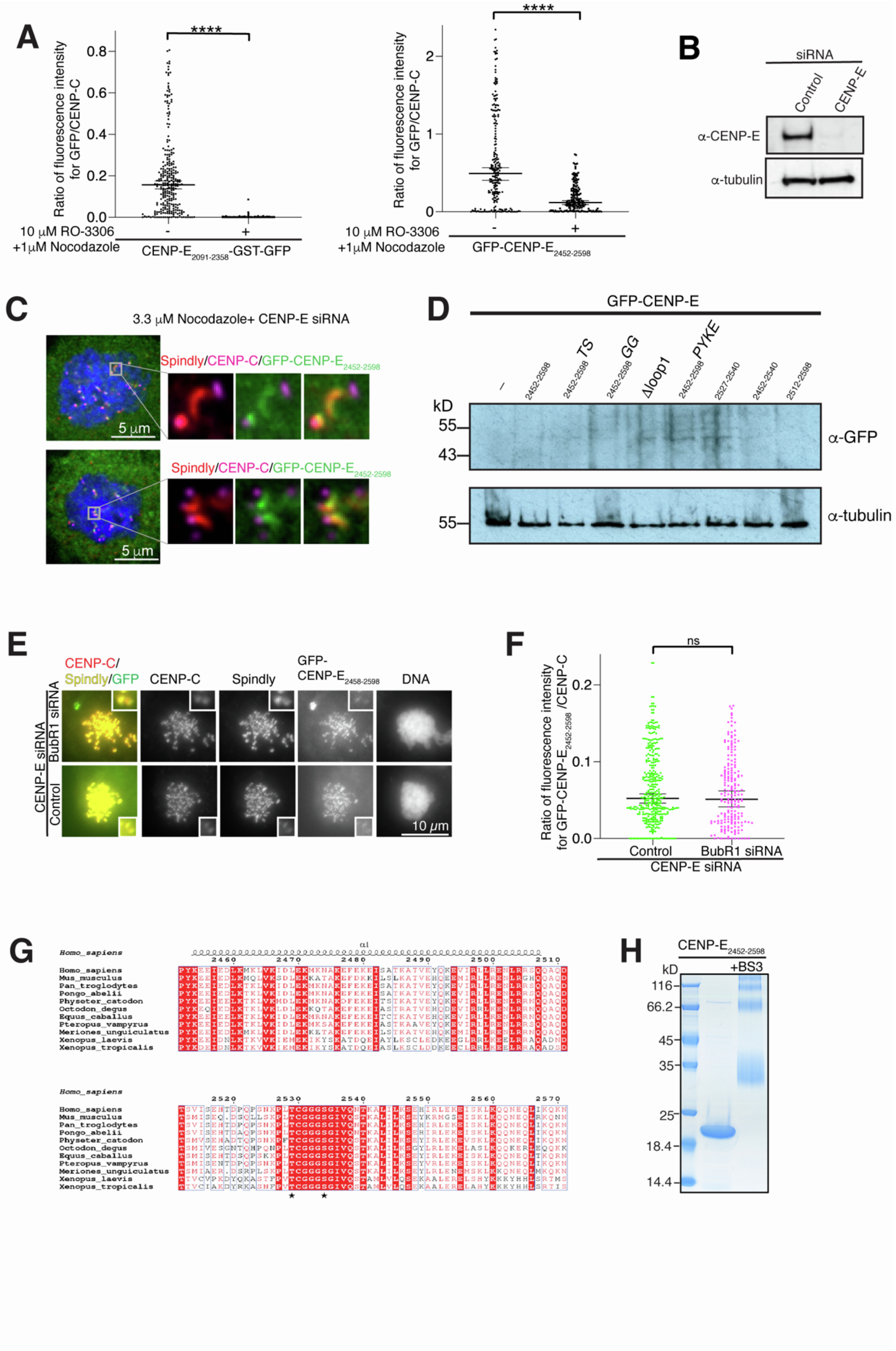

Figure S2. GFP-CENP-E$_{2452\text{-}2598}$ targets to the outer corona of kinetochores. Related to Figure 2. (A) Ratio of fluorescence intensity for GFP/mCherry-CENP-A for cells transfected

with CENP-$E_{2091-2358}$-GST-GFP and GFP-CENP-$E_{2452-2598}$ treated with 1 μM nocodazole before and 30 minutes after incubation with 10 μM CDK1 inhibitor RO-3306. Bars represent median and 95% confidence interval. Unpaired T-test with P ***<0.0001. For CENP-$E_{2091-2358}$-GST-GFP, n=273 and n=255 from 7 cells in the presence of nocodazole and nocodazole+CDK1 inhibitor respectively. For GFP-CENP-$E_{2452-2598}$, n=219 and n=183 from 5 cells in the presence of nocodazole and nocodazole+CDK1 inhibitor respectively. (B) Western blot for cells after control or CENP-E siRNA treatment probed for CENP-E. Tubulin is presented as a loading control. (C) Representative immunofluorescence images of HeLa cells after CENP-E siRNA depletion, transfected with GFP-CENP-$E_{2452-2598}$ and treated with 3.3 μM nocodazole and 10 μM RO-3306, stained for Spindly and CENP-C. Scalebar: 5 μm. (D) Western blot of the transiently-transfected HeLa cells expressing GFP-CENP- $E_{2452-2598}$ and mutants probed for GFP, for experiments presented in Figure 2. Tubulin is presented as a loading control. (E) Representative images of HeLa cells after siRNA-mediated depletion of CENP-E, or CENP-E and BubR1, transfection with GFP-CENP-$E_{2452-2598,}$ and treatment with nocodazole and RO-3306. The cells were stained for CENP-C, Spindly and DNA. Scalebar: 10 μm. (F) Graph quantifying the levels of GFP-CENP-$E_{2452-2598}$ at kinetochores relative to CENP-C after depletion of CENP-E, or CENP-E and BubR1 by siRNA. Bars represent median and 95% confidence interval. An unpaired T-test showed no significant difference between the two conditions. (G) Sequence alignment of the conserved region of the kinetochore-targeting region of CENP-E containing 2452-2571 with mouse, chimpanzee, orangutan, sperm whale, degu, horse, flying fox, gerbil and *Xenopus* CENP-E sequences. Boxed red and blue areas respectively highlight conserved and similar amino acids across all species. Amino acids in red are those with conserved properties in sequence from at least 3 species. The residues mutated in the *TS* mutant are marked with a star (*). (H) Coomassie-stained gel showing CENP-$E_{2452-2598}$ without crosslinking and after crosslinking with BS3.

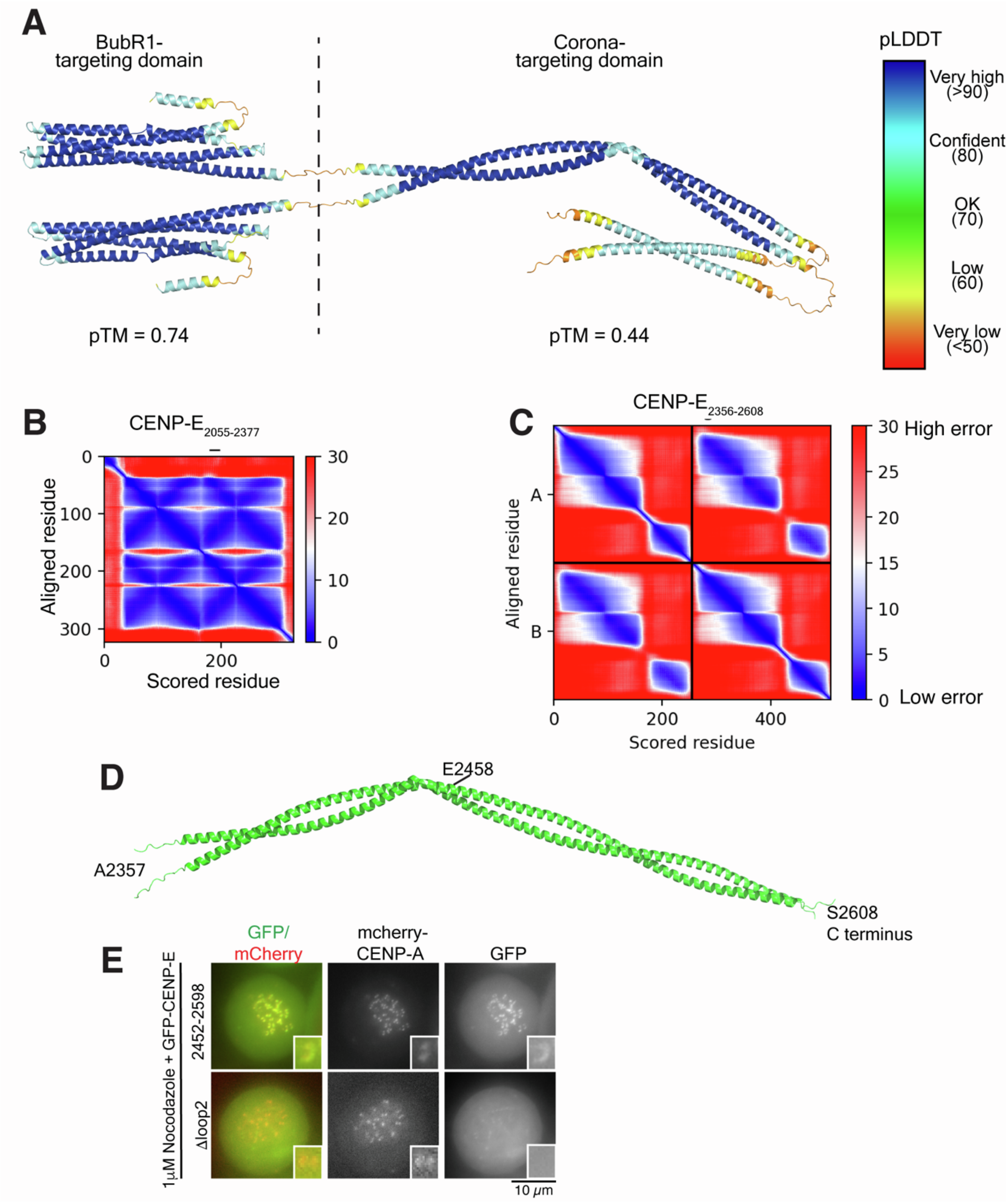

Figure S3. Alphafold-derived structural model of CENP-E$_{2055\text{-}2608}$. Related to Figure 2. (A) Model of CENP-E$_{2055\text{-}2608}$ coloured according to pLDDT scores, between blue (>90) and red (<50). (B, C) Predicted aligned error scores between each amino-acid of the single chain and two chains of the BubR1-targeting domain (CENP-E 2055-2377) and two chains of the corona-targeting domain (CENP-E 2356-2608), respectively (B and C), showing blue (low error) and red (high error) predictions. (D) Alphafold model of the CENP-E domain Δloop2 (2513-2537 deleted). (E) Representative live-cell images of HeLa cells expressing mCherry-CENP-A and

transfected with GFP-CENP-$E_{2452\text{-}2598}$ or GFP-CENP-E Δloop2 and treated with 1 μM nocodazole for 2 hours.

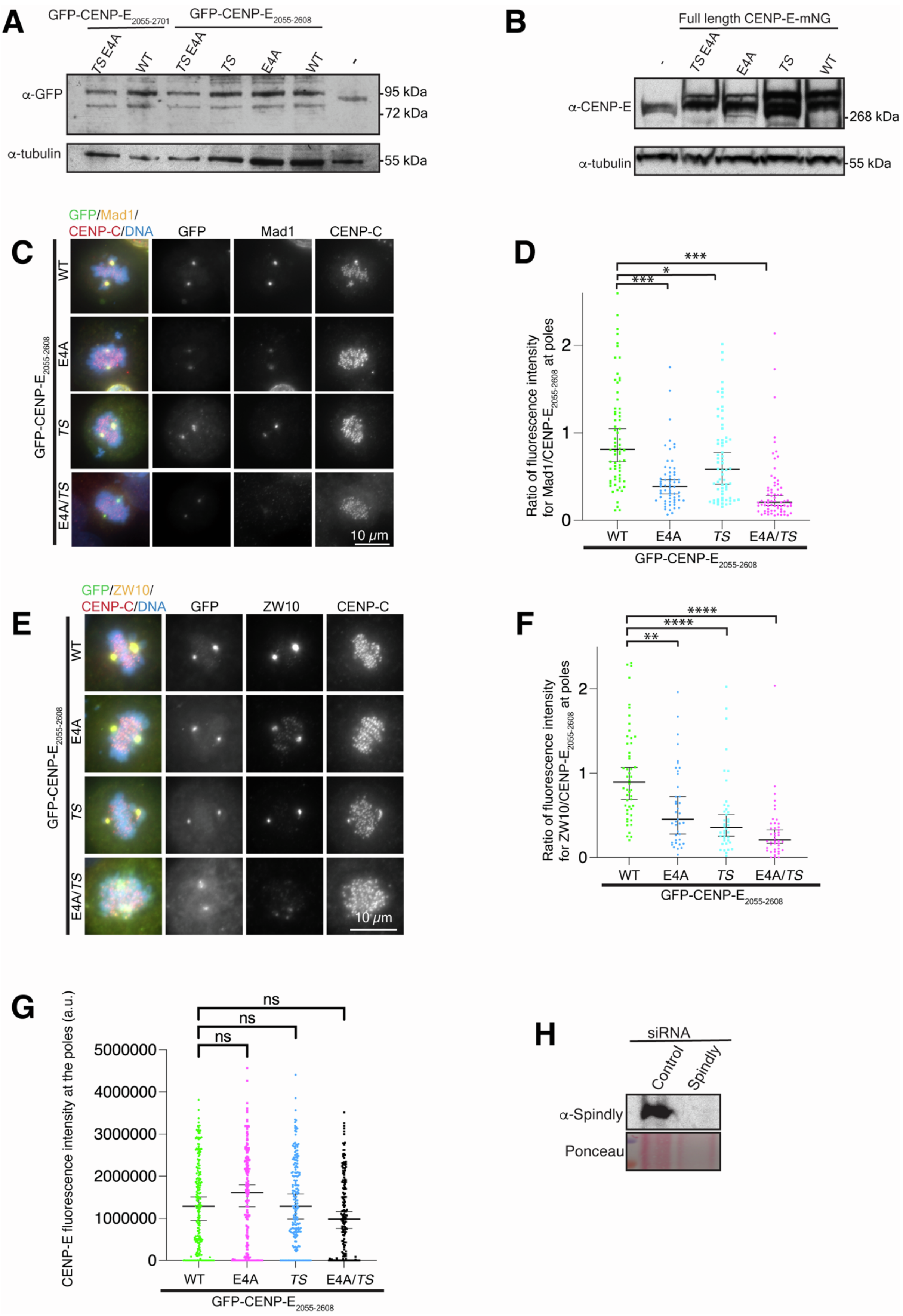
A
GFP-CENP-E2055-2701
GFP-CENP-E2055-2608
TS E4A
WT
TS E4A
TS
E4A
WT
-
α-GFP
95 kDa
72 kDa
α-tubulin
55 kDa
B
Full length CENP-E-mNG
-
TS E4A
E4A
TS
WT
α-CENP-E
268 kDa
α-tubulin
55 kDa
C
GFP/Mad1/
CENP-C/DNA
GFP
Mad1
CENP-C
GFP-CENP-E2055-2608
WT
E4A
TS
E4A/TS
10 μm
D
Ratio of fluorescence intensity for Mad1/CENP-E2055-2608 at poles
***
*
***
WT
E4A
TS
E4A/TS
GFP-CENP-E2055-2608
E
GFP/ZW10/
CENP-C/DNA
GFP
ZW10
CENP-C
GFP-CENP-E2055-2608
WT
E4A
TS
E4A/TS
10 μm
F
Ratio of fluorescence intensity for ZW10/CENP-E2055-2608 at poles
****
****
**
WT
E4A
TS
E4A/TS
GFP-CENP-E2055-2608
G
CENP-E fluorescence intensity at the poles (a.u.)
5000000
4000000
3000000
2000000
1000000
0
ns
ns
ns
WT
E4A
TS
E4A/TS
GFP-CENP-E2055-2608
H
siRNA
Control
Spindly
α-Spindly
Ponceau

Figure S4. characterization of CENP-E cell lines. Related to Figure 3 and Figure 4. (A) Western blot of the Flp-in HeLa cells expressing stably GFP-CENP-$E_{2055-2608}$ and GFP-CENP-$E_{2055-2701}$ mutants after CENP-E siRNA-depletion and doxycycline-induced expression, probed for GFP. Tubulin is presented as a loading control. (B) Western blot of U2OS cells expressing stably full-length CENP-E-mNG wild type and mutants after CENP-E siRNA-depletion and doxycycline-induced expression, probed for CENP-E. Tubulin is presented as a loading control. (C) Representative immunofluorescence images of HeLa cells transfected with GFP-CENP-$E_{2055-2608}$ wild type and mutants, stained for Mad1, CENP-C and DNA. Scalebar: 10 μm. (D) Scatter plot showing ratio of fluorescence intensity of Mad1 to GFP-CENP-$E_{2055-2608}$ wild type or mutants at spindle poles (WT n=70, E4A n=62, *TS* n=66 and E4A *TS* n=70). Bars represent median and 95% confidence interval. Kruskal-Wallis test with P ***<0.0001. The experiment was repeated 3 times. (E) Representative immunofluorescence images of HeLa cells transfected with GFP-CENP-$E_{2055-2608}$ wild type and mutants, stained for ZW10, CENP-C and DNA. (F) Scatter plot showing normalized ratio of fluorescence intensity of ZW10 to GFP-CENP-$E_{2055-2608}$ wild type and mutants at spindle poles (WT n=50, E4A n=39, *TS* n=40 and E4A *TS* n=44). Bars represent the median and 95% confidence interval. Kruskal-Wallis test with P-value **<0.01, ****<0.0001. The experiment was repeated 3 times. (G) Scatter plot showing fluorescence intensity of GFP-CENP-$E_{2055-2608}$ wild type or mutants at spindle poles for datapoints analyzed in Figure 4 and S4C, D (WT n=208, E4A n=190, *TS* n=196 and E4A *TS* n=160). Bars represent median and 95% confidence interval. A Kruskal-Wallis test shows non-significant P-values >0.05. (H) Western blot for cells after control and Spindly siRNA treatment probed for Spindly. Ponceau staining is shown as a loading control.

	CENP-E (PDB 8OWI)
**Data collection**	
Space group	$P4_22_12$
Cell dimensions	
a, *b*, *c* (Å)	84.02, 84.02, 65.02
α, β, γ (°)	90, 90, 90
Resolution (Å)	51.42 – 2.14 (2.34 – 2.14)*
Diffraction limits (Å) and ellipsoidal principal axes	2.572 (1, 0, 0) **a***<br>2.572 (0, 1, 0) **b***<br>2.043 (0, 0, 1) **c***
Eigenvalues and corresponding eigenvectors of anisotropy tensor	76.71 (1, 0, 0) **a***<br>76.71 (0, 1, 0) **b***<br>31.02 (0, 0, 1) **c***
R_{meas}	0.129 (2.897)
R_{pim}	0.026 (0.563)
$I / \sigma(I)$	17.9 (1.3)
$CC_{1/2}$	1.000 (0.565)
Completeness (spherical) (%)	68.8 (15.0)
Completeness (ellipsoidal) (%)	94.4 (76.5)
Redundancy	24.8 (26.0)
**Refinement**	
Resolution (Å)	43.86 – 2.14
No. reflections	9178
R_{work} / R_{free}	0.2161/0.2455
No. atoms	971
Protein	919
Ligand/ion	0
Water	52
B-factors	53.97
Protein	54.01
Ligand/ion	N/A
Water	53.19
R.m.s. deviations	
Bond lengths (Å)	0.004
Bond angles (°)	0.574

*Values in parentheses are for highest-resolution shell.

Table S1. Data collection, phasing and refinement statistics for the structure of CENP-$E_{2454\text{-}2494}$. Related to Figure 2.

Name	Construct	Vector
GFP-CENP-E $_{2055-2608}$	GFP- CENP-E $_{2055-2608}$	pBabe blasticidin
GFP-CENP-E $_{2452-2598}$	GFP- CENP-E $_{2452-2598}$	pBabe blasticidin
GFP-CENP-E $_{2452-2598}$ ∆loop1	GFP- CENP-E $_{2452-2598}$ ∆loop KT binding domain (2527-2538)	pBabe blasticidin
GFP-CENP-E $_{2527-2540}$	GFP- CENP-E $_{2527-2540}$	pBabe blasticidin
GFP-CENP-E $_{2452-2540}$	GFP- CENP-E $_{2452-2540}$	pBabe blasticidin
GFP-CENP-E $_{2512-2598}$	GFP- CENP-E $_{2512-2598}$	pBabe blasticidin
GFP-CENP-E $_{2452-2598}$ *TS*	GFP- CENP-E $_{2452-2598}$ T2529 S2534 mutated to AA	pBabe blasticidin
GFP-CENP-E $_{2452-2598}$ *GG*	GFP- CENP-E $_{2452-2598}$ GG 2531-2532 mutated to NE	pBabe blasticidin
GFP-CENP-E$_{2452-2598}$ *PYKE*	GFP-CENP-E$_{2452-2598}$ PYKE 2452-2455 mutated to AAAA	pBabe blasticidin
CENP-E$_{2091-2358}$-GST-GFP	CENP-E$_{2091-2358}$-GST-GFP	pBabe blasticidin
mCherry-CENP-E$_{2055-2608}$	mCherry-CENP-E $_{2055-2608}$	pBabe puromycin
CENP-E 2091-2450 GFP	CENP-E 2091-2450 GFP	pBabe blasticidin
GFP-CENP-E$_{2055-2608}$ 4E	GFP- CENP-E $_{2055-2608}$ E2313, 2316, 2318, 2319 mutated to A	pcDNA5 FRT TO
GFP-CENP-E $_{2055-2608}$	GFP- CENP-E $_{2055-2608}$	pcDNA5 FRT TO
GFP-CENP-E $_{2452-2598}$	GFP- CENP-E $_{2452-2598}$	pcDNA5 FRT TO
GFP-CENP-E$_{2055-2608}$ *TS*	GFP-CENP-E $_{2055-2608}$ T2529 S2534 mutated to AA	pcDNA5 FRT TO
GFP-CENP-E$_{2055-2608}$ 4E *TS*	GFP-CENP-E $_{2055-2608}$ T2529 S2534 mutated to AA and E2313, 2316, 2318, 2319 mutated to A	pcDNA5 FRT TO
GFP-CENP-E $_{2452-2598}$ ∆loop2	GFP-CENP-E $_{2452-2598}$ ∆loop2 (2516-2532)	pcDNA5 FRT TO
GFP-CENP-E $_{2055-2701}$ 4E *TS*	GFP-CENP-E $_{2055-2701}$ T2529 S2534 mutated to AA and E2313, 2316, 2318, 2319 mutated to A	pcDNA5 FRT TO
GFP-CENP-E $_{2055-2701}$	GFP-CENP-E $_{2055-2701}$	pcDNA5 FRT TO
His$_6$-CENP-E$_{2452-2598}$	His$_6$-CENP-E$_{2452-2598}$	pET3aTr
CENP-E-mNG	Full-length CENP-E mNeonGreen	pLenti
CENP-E-mNG 4E	Full-length CENP-E mNeonGreen E2313, 2316, 2318, 2319 mutated to A	pLenti
CENP-E-mNG *TS*	Full-length CENP-E mNeonGreen T2529 S2534 mutated to AA	pLenti
CENP-E-mNG 4E *TS*	Full-length CENP-E mNeonGreen T2529 S2534 mutated to AA and E2313, 2316, 2318, 2319 mutated to A	pLenti

Table S2. Table summarizing the plasmids used in this study. Related to STAR methods.
